# Multi-class glioma segmentation on real-world data with missing MRI sequences: comparison of three deep learning algorithms

**DOI:** 10.1038/s41598-023-44794-0

**Published:** 2023-11-02

**Authors:** Hugh G. Pemberton, Jiaming Wu, Ivar Kommers, Domenique M. J. Müller, Yipeng Hu, Olivia Goodkin, Sjoerd B. Vos, Sotirios Bisdas, Pierre A. Robe, Hilko Ardon, Lorenzo Bello, Marco Rossi, Tommaso Sciortino, Marco Conti Nibali, Mitchel S. Berger, Shawn L. Hervey-Jumper, Wim Bouwknegt, Wimar A. Van den Brink, Julia Furtner, Seunggu J. Han, Albert J. S. Idema, Barbara Kiesel, Georg Widhalm, Alfred Kloet, Michiel Wagemakers, Aeilko H. Zwinderman, Sandro M. Krieg, Emmanuel Mandonnet, Ferran Prados, Philip de Witt Hamer, Frederik Barkhof, Roelant S. Eijgelaar

**Affiliations:** 1 Centre for Medical Image Computing (CMIC), University College London, London, UK; 2 Neuroradiological Academic Unit, UCL Queen Square Institute of Neurology, University College London, London, UK; 3 Neurosurgical Center Amsterdam, Amsterdam UMC, Vrije Universiteit, Amsterdam, The Netherlands; 4 Department of Neurology & Neurosurgery, University Medical Center Utrecht, Utrecht, The Netherlands; 5 Department of Neurosurgery, St. Elisabeth Hospital, Tilburg, The Netherlands; 6 Neurosurgical Oncology Unit, Department of Oncology and Hemato-Oncology, Università degli Studi di Milano, Milan, Italy; 7 Department of Neurological Surgery, University of California, San Francisco, CA USA; 8 Department of Neurosurgery, Medical Center Slotervaart, Amsterdam, The Netherlands; 9 Department of Neurosurgery, Isala Hospital, Zwolle, The Netherlands; 10 Department of Biomedical Imaging and Image-Guided Therapy, Medical University Vienna, Vienna, Austria; 11 Department of Neurological Surgery, Stanford University, Stanford, USA; 12 Department of Neurosurgery, Northwest Clinics, Alkmaar, The Netherlands; 13 Department of Neurosurgery, Medical University Vienna, Vienna, Austria; 14 Department of Neurosurgery, Medical Center Haaglanden, The Hague, The Netherlands; 15 Department of Neurosurgery, University of Groningen, University Medical Center Groningen, Groningen, The Netherlands; 16 Department of Clinical Epidemiology and Biostatistics, Academic Medical Center, Amsterdam, The Netherlands; 17 TUM-Neuroimaging Center, Klinikum rechts der Isar, Technische Universität München, Munich, Germany; 18 Department of Neurosurgery, Klinikum rechts der Isar, Technische Universität München, Munich, Germany; 19 Department of Neurosurgery, Lariboisière Hospital, APHP, Paris, France; 20 Department of Neuroinflammation, Faculty of Brain Sciences, Queen Square MS Centre, UCL Institute of Neurology, University College London, London, UK; 21 e-Health Center, Universitat Oberta de Catalunya, Barcelona, Spain; 22 Radiology & Nuclear Medicine, VU University Medical Center, Amsterdam, the Netherlands

**Keywords:** CNS cancer, Brain imaging, Cancer imaging

## Abstract

This study tests the generalisability of three Brain Tumor Segmentation (BraTS) challenge models using a multi-center dataset of varying image quality and incomplete MRI datasets. In this retrospective study, DeepMedic, no-new-Unet (nn-Unet), and NVIDIA-net (nv-Net) were trained and tested using manual segmentations from preoperative MRI of glioblastoma (GBM) and low-grade gliomas (LGG) from the BraTS 2021 dataset (1251 in total), in addition to 275 GBM and 205 LGG acquired clinically across 12 hospitals worldwide. Data was split into 80% training, 5% validation, and 15% internal test data. An additional external test-set of 158 GBM and 69 LGG was used to assess generalisability to other hospitals’ data. All models’ median Dice similarity coefficient (DSC) for both test sets were within, or higher than, previously reported human inter-rater agreement (range of 0.74–0.85). For both test sets, nn-Unet achieved the highest DSC (internal = 0.86, external = 0.93) and the lowest Hausdorff distances (10.07, 13.87 mm, respectively) for all tumor classes (*p* < 0.001). By applying Sparsified training, missing MRI sequences did not statistically affect the performance. nn-Unet achieves accurate segmentations in clinical settings even in the presence of incomplete MRI datasets. This facilitates future clinical adoption of automated glioma segmentation, which could help inform treatment planning and glioma monitoring.

## Introduction

Clinically accurate segmentation and longitudinal volumetric analysis of glioma are helpful in treatment planning and response monitoring^1,2^. Volumetric analyses are not commonly used in clinical practice and are generally limited to crude 2D measurements in clinical trials. While this is the current standard for treatment response evaluation in trials^3^, poor prognosis and heterogeneous treatment response encourage quantitative analysis of tumors, especially for glioma due to their varied morphometry and infiltrative nature^4–7^. It is these two characteristics of glioma, along with heterogenous contrast enhancement, that complicate their manual delineation and further highlight the need for automated segmentation protocols in the clinical setting^8–10^. Indeed, baseline imaging and volumetric measurements are of particular importance to neurosurgeons and radiotherapists because tumor volume and functional anatomy are key factors for both risk and prognostic assessment of patients^11,12^.

The VASARI features have illustrated the importance of extracting such quantitative measures, but automation of segmentation and subsequent feature extraction is needed to enable widespread application^13,14^. Automated quantification could provide improvements in reporting time, treatment response monitoring, and overall efficiency across a neuroradiological service, but is dependent upon technical and clinical validation of the methods^15–17^. Deep learning has emerged as the preferred method for automated tumor segmentation^6,18–21^. Ideally, the clinical environment requires a fast algorithm that is robust to scanner variation and missing MRI sequences.

Since 2012, the annual Brain Tumor Segmentation (BraTS) Challenge has compared the performance of numerous AI-driven glioma segmentation algorithms^18,22^. However, these algorithms are trained and assessed on a highly curated dataset optimised for quality: each subject has a complete dataset of high-quality pre- and post-contrast T1-weighted (T1w and T1c, respectively), T2-weighted (T2w), and T2-weighted fluid-attenuated inversion recovery (FLAIR) images, which does not accurately reflect the realities of clinically-acquired MRI data. For example, a recent study using a model (DeepMedic) trained exclusively on BraTS data, achieved a median Dice similarity coefficient (DSC) of 0.81 on BraTS test data but only 0.49 on external clinical data ^23^.

The aim of the current study was to determine the performance and generalisability of three of the highest-performing models at recent BraTS challenges^24–26^ on real-world clinical data. Models have been trained with both BraTS data and another multi-centre dataset obtained from 12 different hospitals worldwide: the PICTURE project (www.pictureproject.nl)^27–31^. An external test set comprised of PICTURE data from hospitals not used in the training and validation phases was employed to assess the clinical applicability and determine the need for retraining models on a hospital’s own data. Furthermore, we use sparsified training, to account for missing sequences^23^, and assessed performance in patients with incomplete MRI datasets.

## Materials and methods

All patients provided informed consent and data were obtained and anonymized according to the General Data Protection Regulation and Health Insurance Portability and Accountability Act. Local Institutional Review Board approval was obtained for all primary studies. For the the VU medical center Amsterdam the institutional review board approved of the experiments in this study under case nr. 2014.336. Of the patients involved in the current study, 40 were previously studied in an inter-rater agreement study by Visser et al.^29^. The 275 Glioblastoma patients from the PICTURE dataset were previously used in a study focused on robust tumor core segmentation in glioblastoma patients Eijgelaar et al.^23^. The study was carried out in concordance with the Checklist for Artificial Intelligence in Medical Imaging (CLAIM) guidelines ^32^.

### BraTS and PICTURE Datasets

We used manual segmentations of preoperative imaging of 1251 gliomas (unspecified mix of GBM and LGG) from the BraTS 2021 dataset and 275 GBM and 205 LGG (median age, 63.7 IQR [54.3–72.0] years; median survival, 323 [142–609] days; surgery extent: 348 resections, 83 biopsies, 49 unknown) from the PICTURE project. The PICTURE dataset was collected across 12 hospitals worldwide, all patients of at least 18 years old with a newly-diagnosed LGG, or GBM at first-time surgery between 1/1/2012 and 12/31/2013 were included. Since the PICTURE data was collected in 2012 and 2013, the classification of GBM and LGG was in line with WHO 2007 criteria. Demographics for the PICTURE dataset are documented in Appendix 1 of the supplementary material.

### Missing scans

**Figure 1:**
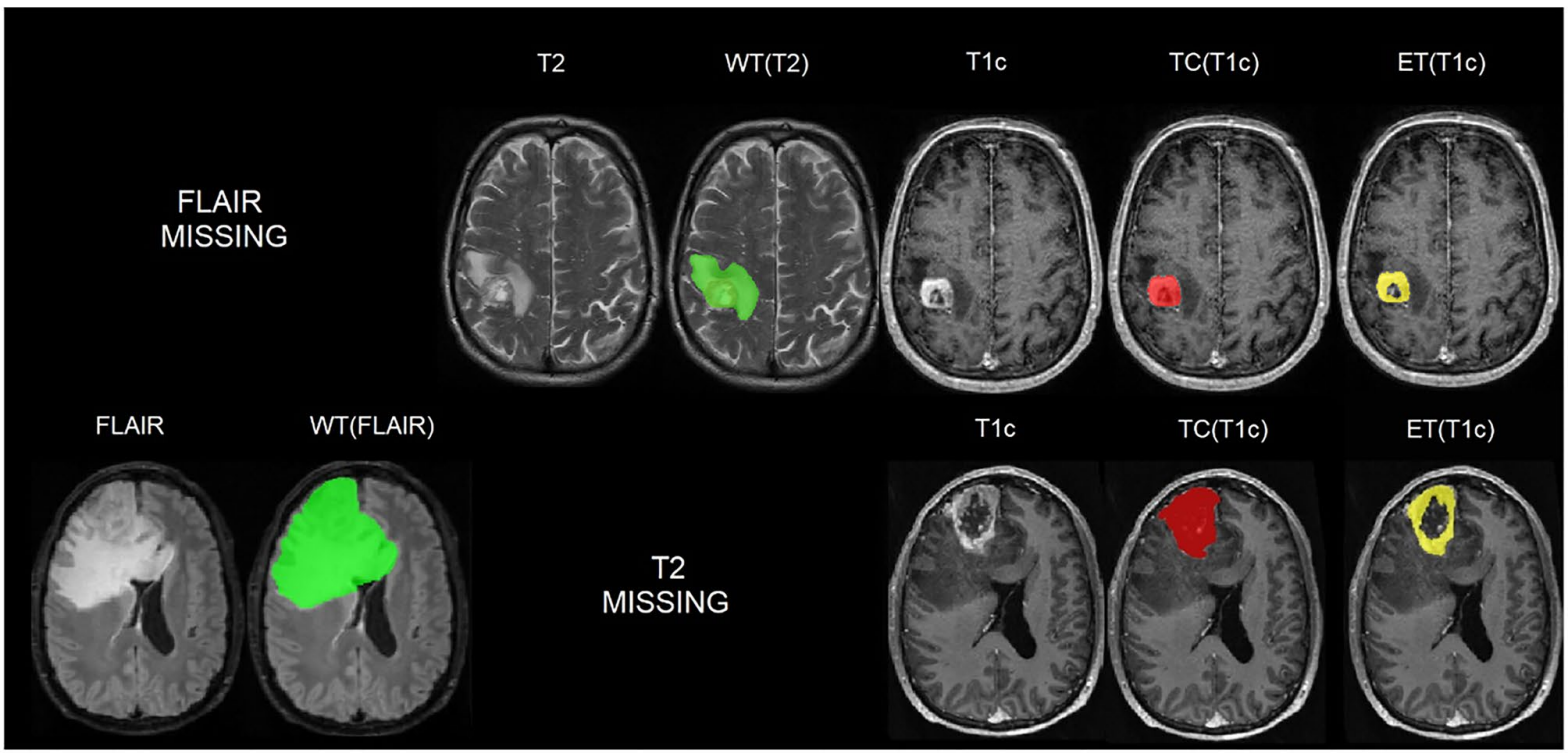
Sample images of PICTURE dataset. Ground truth manual segmentation for a GBM patient with missing FLAIR scan (top row) and one with missing T2w (bottom row), see “Model training and testing” for details of sparsified training which is used to account for missing sequences. Whole Tumor (WT) in green. The WT defines the full extent of the tumor, including the tumor core and oedema, indicated by hyperintensity on FLAIR and T2w. Tumor Core (TC) in red. The TC is the main body of the tumor and most likely area of resection. The TC includes the enhancing tumor (ET) and necrosis. The ET is shown in yellow.

Both datasets contain pre-operative T1w, T1c, T2w, and FLAIR images. However, in the PICTURE dataset some patients had missing sequences, see Table 1 for a breakdown and Fig. 1 for examples of subjects with a missing FLAIR or T2. Only patients with at least T1c and either T2w or FLAIR were included to be able to manually segment all tissue classes. Out of 1731 total cases, there were 204 missing pre-contrast T1w, 186 missing T2w, and 19 missing FLAIR, see “Model training and testing” for details of the sparsified training used to account for missing sequences.

**Table 1:** Breakdown of data used in this study from the BraTS and PICTURE datasets (https://www.pictureproject.nl), and missing data totals from each hospital, as well as a breakdown of the train, validation, test, and external test sets.

Data source	Tumor type	Dataset type	Group	Total	T1w missing	T2w missing	FLAIR missing
BraTS	GBM + LGG n = 1251	Train n = 1000	BraTS	1000	0	0	0
		Validation n = 63	BraTS	63	0	0	0
		Test n = 188	BraTS	188	0	0	0
PICTURE	GBM n = 275	Train n = 95	Hospital 1	52	6	0	3
			Hospital 2	43	1	1	0
		Validation n = 7	Hospital 1	3	0	0	0
			Hospital 2	4	0	1	0
		Test n = 15	Hospital 1	8	0	0	0
			Hospital 2	7	1	0	0
		External test set n = 158	Hospital 3	4	3	1	2
			Hospital 4	15	1	12	0
			Hospital 5	1	0	0	0
			Hospital 6	1	0	0	0
			Hospital 7	1	0	0	0
			Hospital 8	23	13	0	0
			Hospital 9	19	3	0	7
			Hospital 10	8	0	2	3
			Hospital 11	86	2	1	0
	LGG n = 205	Train n = 107	Hospital 2	83	78	78	0
			Hospital 12	14	3	2	0
			Hospital 3	10	10	10	0
		Validation n = 6	Hospital 2	4	4	4	0
			Hospital 3	2	2	2	0
		Test n = 23	Hospital 2	17	17	17	0
			Hospital 12	4	3	2	1
			Hospital 3	2	2	2	0
		External test set n = 69	Hospital 9	13	0	0	0
			Hospital 11	56	55	51	3
Total				1731	204	186	19

### Pre-processing

T1w, T2w, and FLAIR images were rigidly registered to the T1c image. Subsequently, the T1c was registered to the SRI24 atlas (https://www.nitrc.org/projects/sri24/)^33^ using an affine transformation. The same transform was applied to the other MR sequences (T2w, FLAIR, T1w). All modalities were resampled to 1mm isotropic voxels in the SRI24 atlas space, the rigid and affine registrations were applied using a single interpolation step. All registrations and resampling were conducted using the Advanced Normalization Tools (ANTs)^34^. N4 bias field correction^35^ was used and skull stripping was performed with the “HD-bet” algorithm (https://github.com/MIC-DKFZ/HD-BET) ^36^.

### Manual segmentations

For the PICTURE data, 275 GBM and 205 LGG cases were manually segmented into 3 classes consistent with the BraTS challenges – whole tumor (WT), tumor core (TC), and enhancing tumor (ET), see Fig. 1. The WT defines the full extent of the tumor, including the tumor core and oedema, indicated by hyperintensity on FLAIR and T2w. The TC is the main body of the tumor and most likely area of resection. The TC includes the enhancing tumor (ET) and necrosis.

Manual segmentations were carried out according to the VASARI Research Project (https://wiki.cancerimagingarchive.net/display/Public/VASARI+Research+Project). One rater (HP) with 9 years of brain MRI manual segmentation experience performed segmentations under the supervision and approval of an expert neuroradiologist (FB), using the semiautomatic SmartBrush tool (BrainLab, Feldkirchen, Germany). The rater’s performance was in line with experts^29^. All segmentations were exported on the T1c image. The segmentation was resampled to SRI24 atlas space using the same transform from the T1c to SRI24 registration.

### Quality control

Visual quality control checks were carried out for incomplete coverage, skull stripping, registration errors, and incomplete segmentations. Overview images were generated to facilitate quality control. The images show the same axial, sagittal, and coronal view for all patients to assess the registration quality, as well as an axial view of the center of the tumor to verify the segmentation. Seven scans were not included due to poor image quality and five due to severe registration errors (as illustrated in Appendix 2).

### Deep learning segmentation models

Three algorithms were selected for this study based on high performance in recent BraTS challenges^18,22,37^, availability of a user-friendly and reproducible implementation online, and the uniqueness of the algorithm, see Table 2.

**Table 2:** Summary of the deep learning algorithms tested in this study.

Name	Description	References
nvNet	A 3D U-net-based architecture using skip-connections, group normalization and variational autoencoder based regularization	^26,51^ (https://ngc.nvidia.com/catalog/resources/nvidia:clara:clara_ai_brain_tumor_pipeline/files?version=0.7.1-2008.1)
DeepMedic	A multi-scale, 3D patch-based fully convolutional classification network. In contrast to U-net, DeepMedic does not have an up-sampling ‘side’. It predicts 1 × 1x1 voxels based on a high- and low-res input of 17 × 17x17 voxel, the low-res input uses a down sampled version of the image	^25,52^ (https://github.com/deepmedic/deepmedic)
nn-Unet (‘no-new-Net’)	A U-net network architecture using a 2D, 3D and a cascaded U-net. Three U-net structures are trained simultaneously, and the best trained model is automatically selected	^24^ (https://github.com/MIC-DKFZ/nnUNet)

### Model training and testing

Models were trained with three-class segmentations (WT, TC, ET) for each tumor. The scans were randomly split in 80% training, 5% validation, and 15% internal test data, see Table 1. Test data was used to assess the performance of each model. Alongside the 15% internal test data, models were further assessed using an external test set of 158 GBM and 69 LGG patients from PICTURE hospitals not included in the training data, herein referred to as the external test set. This helped to gauge the generalisability and determine the future need for retraining algorithms on a new hospital’s unseen data.

In order to address missing sequences in the training data (Table 1), sparsified training was applied for all algorithms^23^. This study showed that performance drops substantially if not all sequences are available. This could be solved by inserting empty (zero-filled) scans in place of missing sequences, see the first column of Fig. 1. During training, the T1w, T2w, and FLAIR were additionally set to zero with independent probabilities of 20%, in line with the estimated frequency of missing sequences in the clinical setting^23^. We used the validation data to confirm convergence, the hyperparameters of all models were kept at the default values, as reported in the associated papers, or as used in the published code repositories (Table 2). All model training and testing was carried out using a machine equipped with an AMD Ryzen 9 3900X 12 core processor, 64GB RAM, and 1 NVIDIA RTX3090 (24GB) graphics processing unit (GPU).

### Model performance assessment

In line with the BraTS challenges, tumor segmentations for each algorithm were assessed using median and inter-quartile range Dice similarity coefficient (DSC)^38^ and Hausdorff distance (HD)^39^ for all experiments. Results were generated using methods described by Taha and Hanbury^40^ and associated software.

### Experiments and statistical analyses

Four separate experiments were performed. In experiment 1, DSC and HD from the internal and external test sets were analyzed separately for each model/tumor class using a paired two-tailed t-test to assess differences between each model. DSC and HD were also compared in the following experiments using independent samples (Welch’s) t-tests: experiment 2—GBMs vs LGGs on the internal and external test set to assess differences in performance on the differing tumor grades; experiment 3—GBMs and LGGs from the internal vs external test sets to assess the change in performance when segmenting external hospital data not previously seen by the models; and experiment 4—GBM and LGG patients with incomplete vs complete imaging datasets in the external test set to assess the change in performance when segmenting patients with incomplete imaging datasets from external hospital data not yet seen by the models. A single Bonferroni correction was applied for each experiment^41^. Outliers in box plots and overall outlier rates for each model and segmentation class were calculated using the IQR × 1.5 rule, i.e. outside [Q1 − 1.5 × IQR; Q3 + 1.5 × IQR]^42,43^.

## Results

### PICTURE dataset segmentations

**Figure 2:**
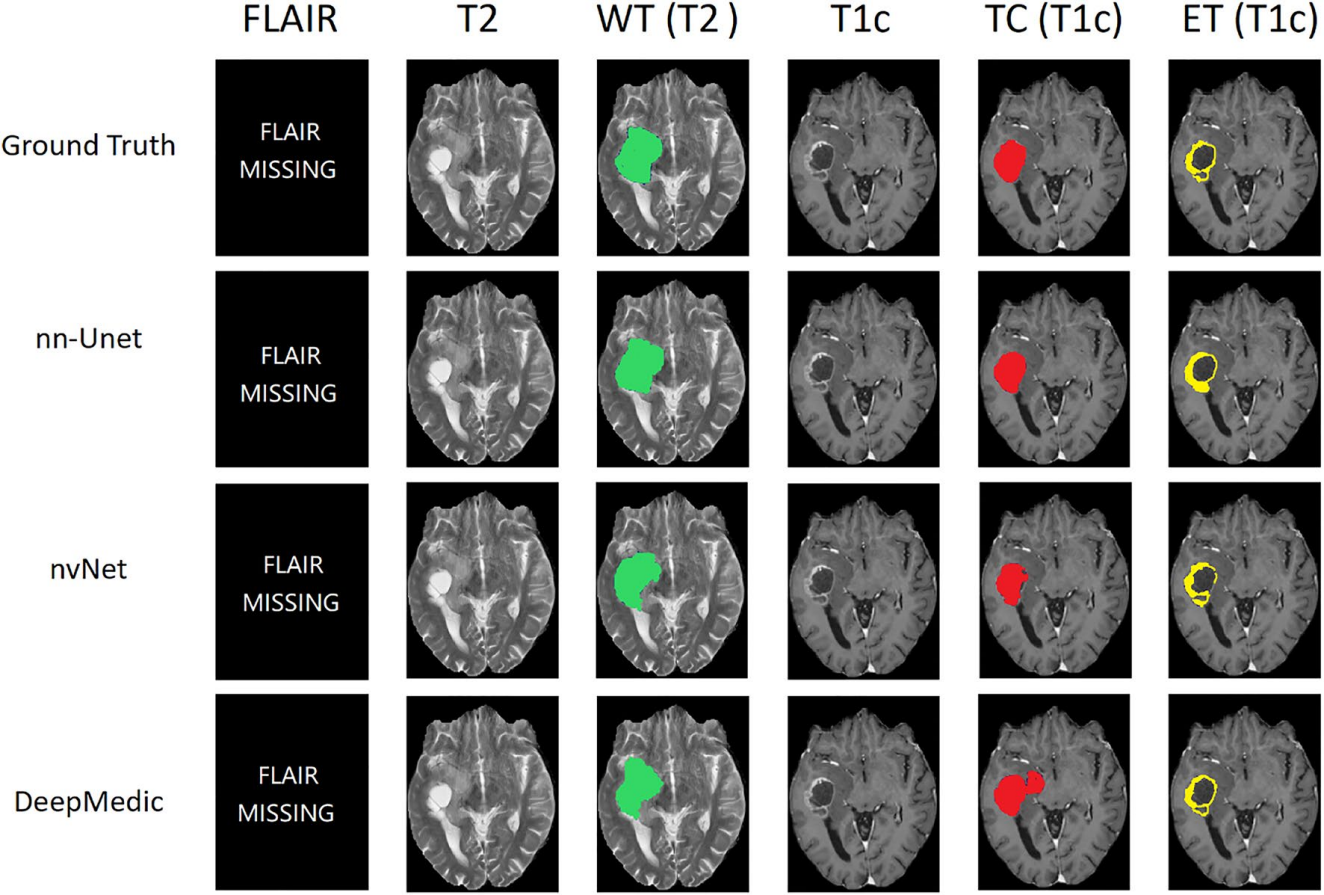
GBM patient from the PICTURE dataset with missing FLAIR scan. Whole Tumor (WT—green) is the full extent of the tumor, including the tumor core, non-enhancing tumor and oedema, indicated by hyperintensity on FLAIR and T2w. Tumor Core (TC—red) is the main body of the tumor and most likely area of resection. The TC includes the enhancing tumor (ET—yellow) and necrosis. DSCs in this case for nn-Unet were WT = 0.93, TC = 0.94, ET = 0.83; nvNet WT = 0.89, TC = 0.92, ET = 0.80; and DeepMedic WT = 0.81, TC = 0.85, ET = 0.81.

See Fig. 2 for ground truth manual segmentation and automated segmentation examples from all three models. See Appendix 3 and 4 for GBM and LGG segmentation contours on a 4 T T1c scan along with two human experts’ manual segmentation, for all tumor classes.

### Experiment 1—Segmentation performance on both test sets—which model achieved the best metrics?

**Figure 3:**
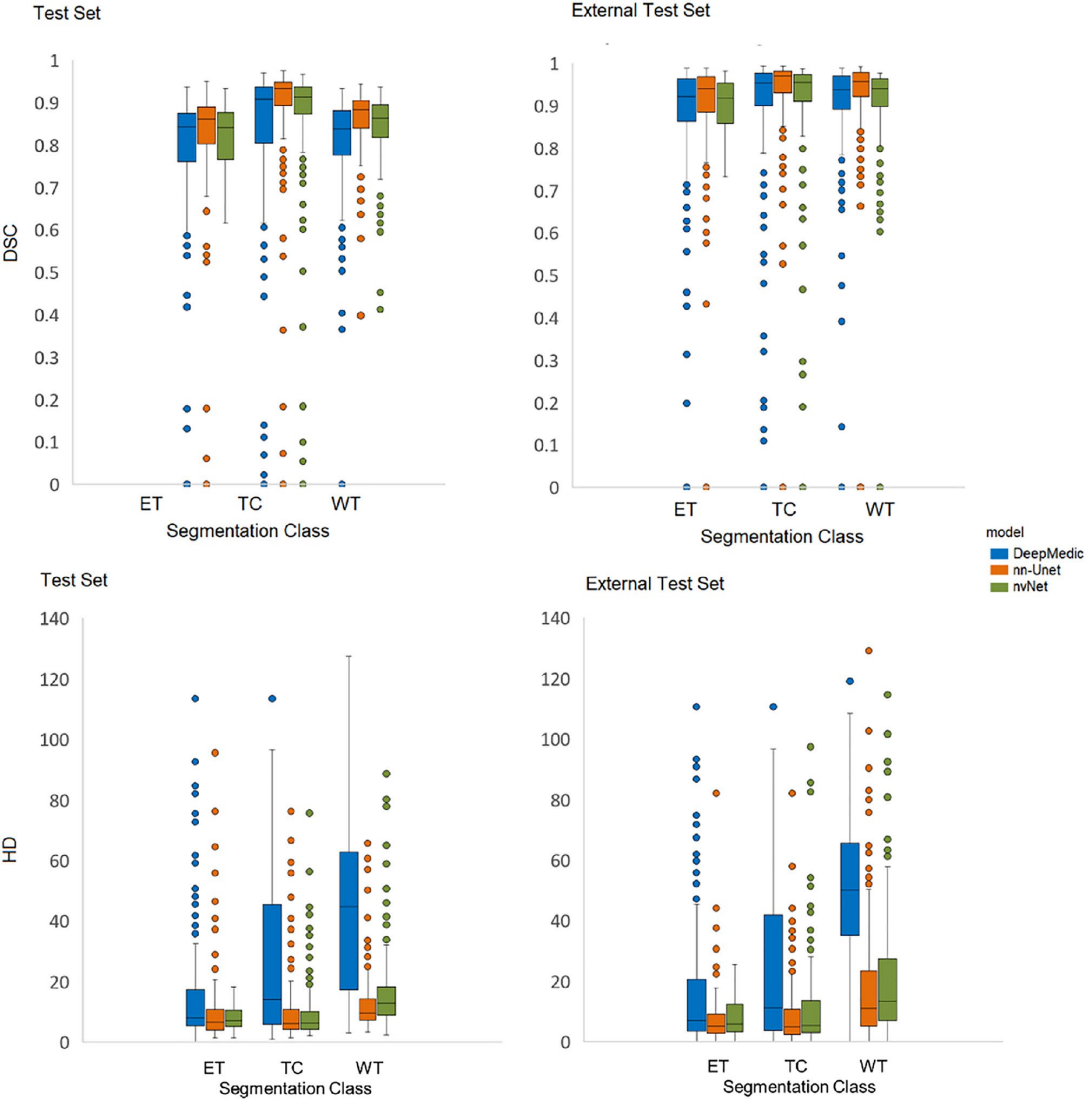
Box plots showing DSC and HD in internal- and external-test sets for all models and tumor classes. Left plots show the test set performance (n = 226) and the right plots show the performance in the external test set (n = 277).

Box plots showing median DSC and HD for all models and tumor classes on the internal and external test sets are presented in Fig. 3. nn-Unet achieved significantly higher DSCs than nvNet and lower HDs than both nvNet and DeepMedic for all tumor classes on both the internal and external test sets (p values < 0.0027). The raw metrics are displayed in Table 3.

**Table 3:** Median DSC and HD for all models and tumor classes on the internal test set GBMs (n = 15) and external GBMs test set (n = 158).

Model	DSC (IQR)			HD (IQR)		
	Internal test set	External test set	t-test, *p*	Internal test set	External test set	t-test, *p*
**Whole tumor (GBM)**						
DeepMedic	0.82 (0.10)	0.84 (0.13)	< 0.001*	58.11 (27.16)	45.08 (44.10)	0.018
nn-Unet	**0.97 (0.09)**	**0.95 (0.10)**	< 0.001*	**7.34 (10.21)**	**9.11 (10.30)**	0.958
nvNet	0.94 (0.11)	0.92 (0.15)	< 0.001*	8.06 (6.93)	12.83 (14.56)	0.087
**Tumor core (GBM)**						
DeepMedic	0.88 (0.09)	0.91 (0.14)	0.013	28.93 (37.26)	13.48 (48.96)	< 0.001*
nn-Unet	**0.97 (0.07)**	**0.96 (0.06)**	0.478	**4.12 (7.41)**	**4.47 (6.47)**	0.374
nvNet	0.96 (0.09)	0.94 (0.14)	0.052	5.00 (4.65)	5.38 (6.48)	0.099
**Enhancing tumor (GBM)**						
DeepMedic	0.76 (0.11)	0.84 (0.10)	< 0.001*	12.53 (16.27)	8.03 (40.38)	0.028
nn-Unet	**0.83 (0.06)**	**0.86 (0.10)**	< 0.001*	**5.14 (4.98)**	**6.44 (5.54)**	0.536
nvNet	0.77 (0.11)	0.84 (0.15)	< 0.001*	6.70 (4.35)	7.03 (5.17)	0.926
**Whole tumor (LGG)**						
DeepMedic	0.88 (0.15)	0.82 (0.18)	0.409	79.11 (75.16)	49.98 (61.10)	0.458
nn-Unet	**0.89 (0.08)**	**0.87 (0.11)**	0.023	**10.68 (5.18)**	**9.82 (7.30)**	0.944
nvNet	0.88 (0.12)	0.85 (0.13)	0.036	17.54 (8.93)	11.83 (7.86)	0.771
**Tumor core (LGG)**						
DeepMedic	0.86 (0.15)	0.80 (0.23)	0.095	32.52 (64.88)	42.48 (60.96)	0.731
nn-Unet	**0.89 (0.07)**	**0.86 (0.11)**	0.029	**10.70 (5.81)**	**9.71 (9.54)**	0.277
nvNet	0.86 (0.11)	0.82 (0.21)	0.049	11.19 (7.65)	15.38 (10.66)	0.240
**Enhancing tumor (LGG)**						
DeepMedic	N/A					
nn-Unet						
nvNet						

Bold font indicates most favourable score in each scenario. Bonferroni adjusted p values at < 0.0027 comparing the performance of models on test set vs external test set were considered significant and are denoted by asterisk, *

### Experiment 2—Segmentation performance on GBM vs LGG

**Figure 4:**
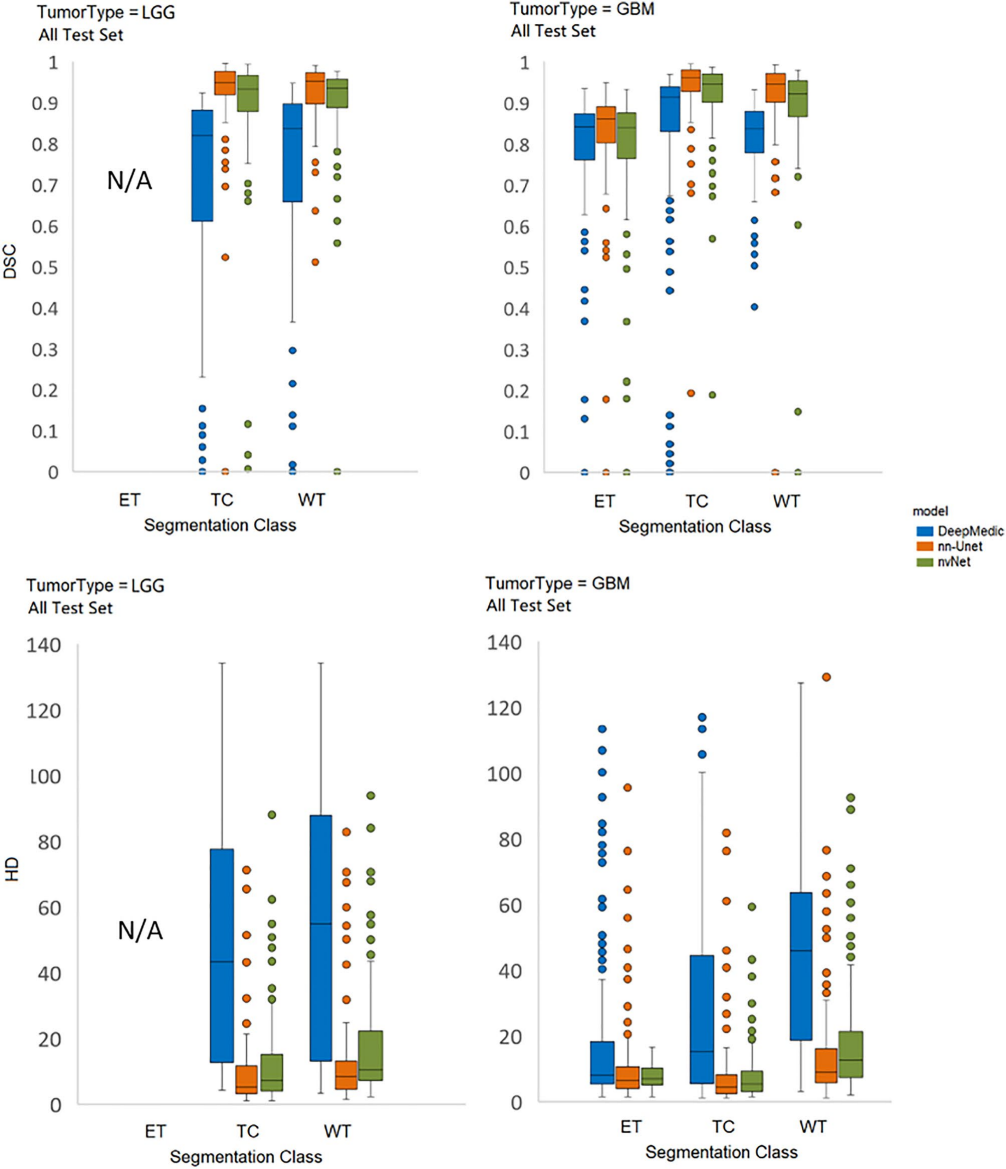
Box plots showing DSC and HD in LGG and HGG patients for all models and tumor classes. upper row shows DSC and the bottom row shows HD for all models and tumor classes on GBM (n = 15 test + 158 external test, on the right) vs LGG (n = 23 internal test + 69 external test, on the left) patients.

Comparing performance between GBM and LGG, nn-Unet continued to provide the best quality results for both tumor grades, statistical comparisons are reported in Table 4. However, overall segmentation performance on the LGG was notably weaker than for GBM across all models, see Fig. 4 for box plots. DeepMedic showed the largest decrease in performance across all tumor classes.

**Table 4:** DSC and HD for all models and tumor classes on all GBM’s (n = 173) and all LGGs (n = 92) in the internal test set plus external test set combined.

Model	DSC (IQR)			HD(IQR)		
	GBMs	LGGs	t-test, *p*	GBMs	LGGs	t-test, *p*
**Whole tumor**						
DeepMedic	0.84 (0.11)	0.83 (0.13)	< 0.001*	45.88 (40.27)	54.83 (53.28)	< 0.001*
nn-Unet	**0.95 (0.09)**	**0.95 (0.12)**	0.054	**8.94 (10.25)**	**8.39 (11.39)**	0.415
nvNet	0.92 (0.10)	0.93 (0.13)	0.189	12.56 (14.56)	10.44 (11.89)	0.226
**Tumor core**						
DeepMedic	0.91 (0.07)	0.82 (0.28)	< 0.001*	15.30 (35.47)	43.39 (58.78)	0.025
nn-Unet	**0.96 (0.07)**	**0.95 (0.24)**	< 0.001*	**4.47 (6.74)**	**5.15 (12.98)**	0.156
nvNet	0.94 (0.08)	0.93 (0.19)	< 0.001*	5.38 (11.12)	7.28 (15.83)	0.012
**Enhancing tumor**						
DeepMedic	0.84 (0.09)	N/A	N/A	8.09 (15.36)	N/A	N/A
nn-Unet	**0.86 (0.06)**	N/A	N/A	**6.40 (5.98)**	N/A	N/A
nvNet	0.84 (0.10)	N/A	N/A	6.96 (7.85)	N/A	N/A

Bold font indicates best score in each scenario. Bonferroni adjusted P values < 0.0027 comparing the performance of models were considered significant and are denoted by asterisk, *

### Experiment 3—GBM segmentation performance on an external test set—do models need to be retrained for new hospital data?

**Figure 5:**
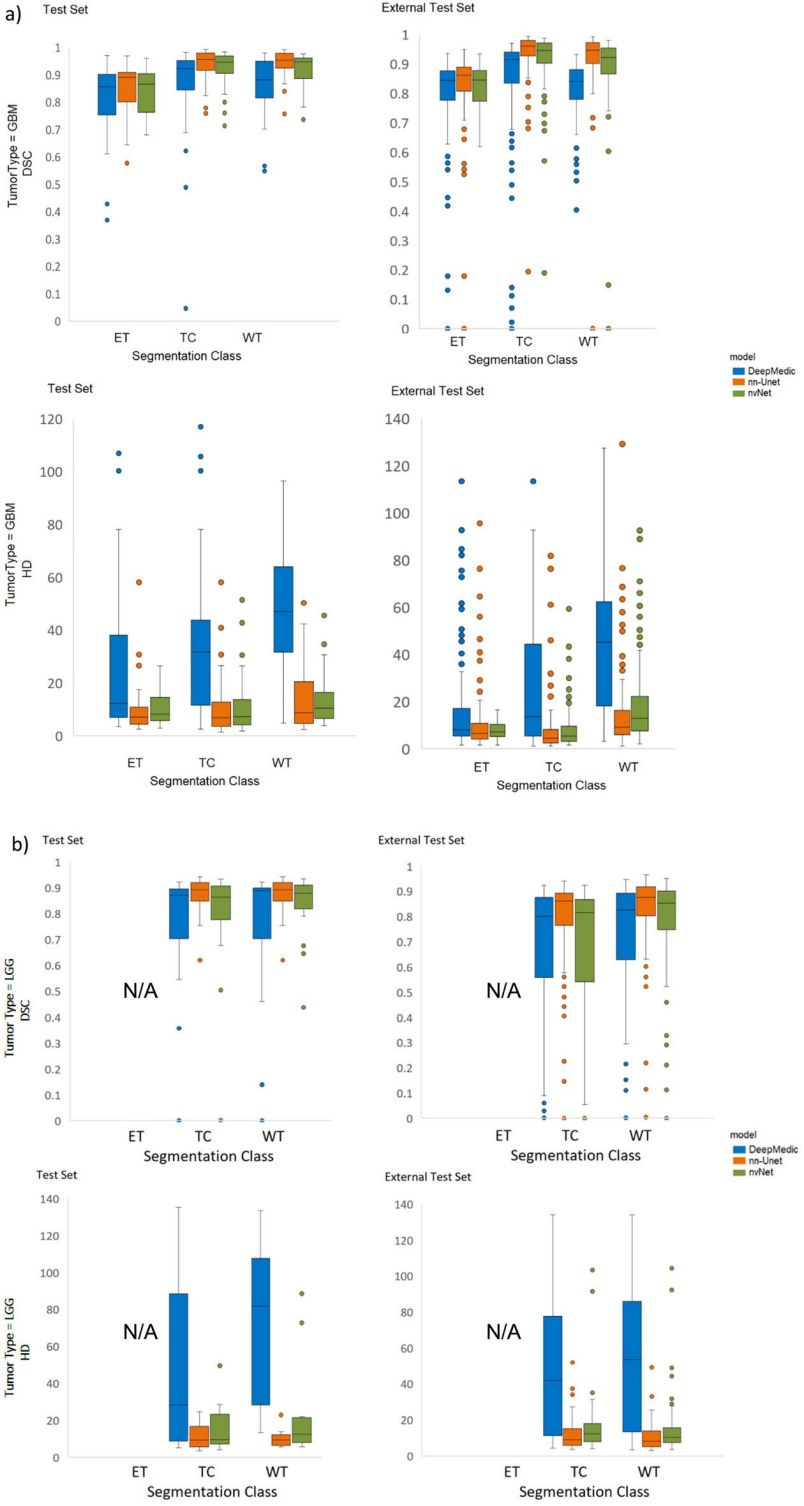
Box plots showing DSC and HD for all models and tumor classes on the internal test set. (**a**) shows GBMs (n = 15 internal test set cases plus 23 BraTS test set GBM cases, upper left plots) and external test set GBMs (n = 158, upper right plots). (**b**) shows LGG (n = 23 internal test, lower left plots + 69 external test, lower right plots).

As shown in experiment 1, nn-Unet produced the most favourable results when compared to the other models on both test sets. Table 3 shows the DSC and HD results for the internal test set (15 GBM, 23 LGG) and the external test set (158 GBM, 69 LGG) comprised of cases from hospitals not included in the training data, see Fig. 5 for box plots. nn-Unet showed the smallest absolute decrease and increase in respectively DSC and HD from the internal to the external test set for GBM WT, (DSC internal: 0.97, external: 0.95, p < 0.001*, HD internal: 7.34, external: 9.11, p = 0.958). All models’ DSC were slightly reduced on WT and TC for both HGG and LGG but remained within clinically-acceptable range^18,22,44^. However, the segmentation performance of ET improved in the external dataset for all models.

### Experiment 4—Effect of missing MRI sequences on segmentation performance

**Figure 6:**
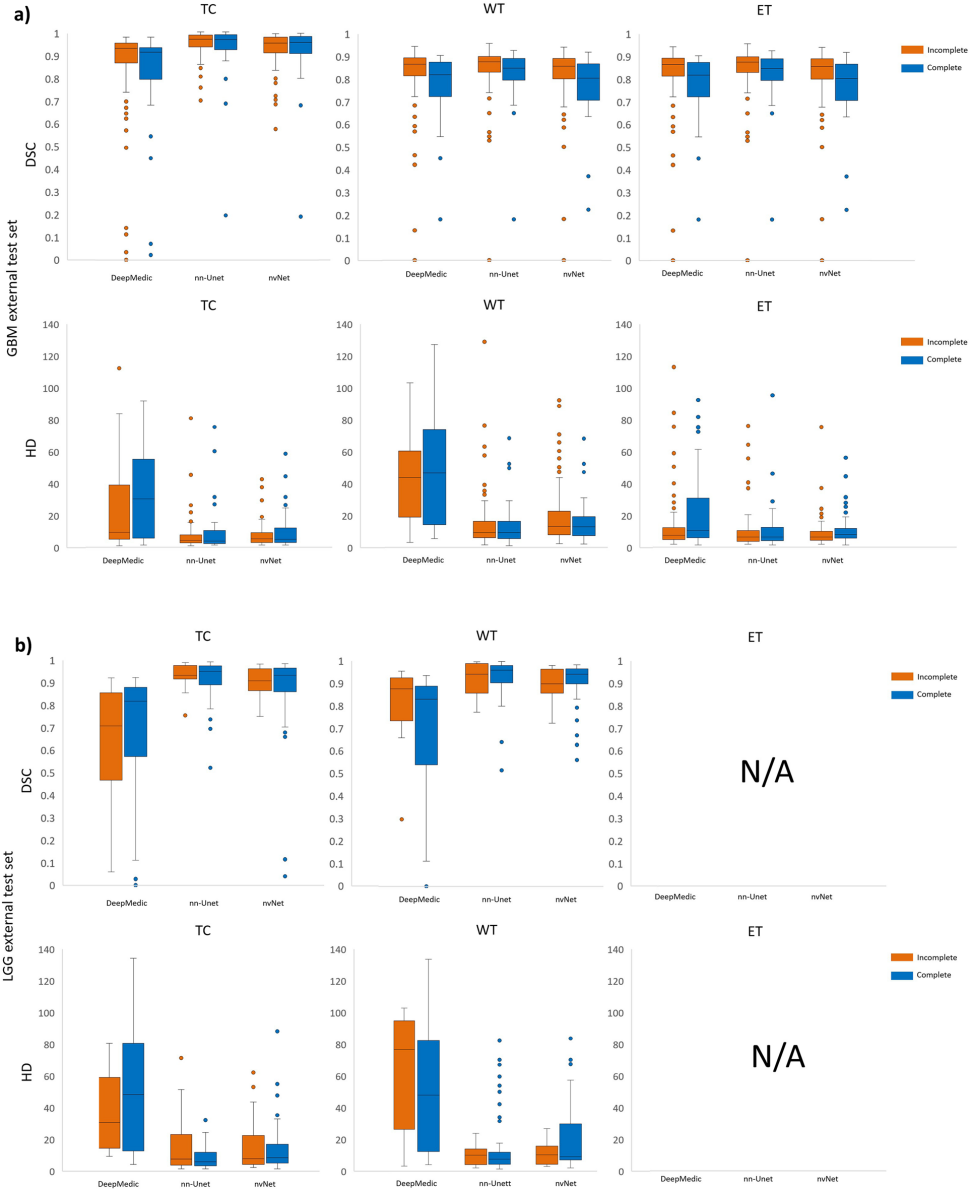
Box plots showing DSC and HD for patients with missing pulse-sequences, and subjects with complete scans. For all models and tumor classes (HGG in panel **a**, LGG in panel **b**) on patients in the external test set with missing pulse-sequences in orange (n = 44GBM + 55LGG) and subjects with complete scans in blue (n = 114GBM + 14LGG).

Box plots showing DSC and HD of GBM and LGG patients with incomplete (44GBM, 55LGG) versus complete (114GBM, 14LGG) scans in the external test set are presented in Fig. 6 and Table 5 separately. For GBM, nn-Unet achieved the highest DSCs and lowest HD for all tumor classes on both incomplete and complete scans, with the exception of nvNet reaching a slightly lower HD on TC for incomplete scans. There were no statistically significant differences between the two groups for all models.

**Table 5:** Median DSC and HD for all models and tumor classes on patients in the external test set with missing sequences (n = 44GBM + 55LGG) and subjects with complete scans (n = 114GBM + 14LGG).

Model	DSC (IQR)						HD (IQR)					
	Incomplete		Complete		*p*		Incomplete		Complete		*p*	
	GBM	LGG	GBM	LGG	GBM	LGG	GBM	LGG	GBM	LGG	GBM	LGG
**Whole tumor**												
DeepMedic	0.80 (0.13)	0.82 (0.35)	0.85 (0.08)	0.87 (0.19)	0.27	0.23	46.69 (62.60)	48.22 (70.09)	43.945 (44.70)	77.06 (68.75)	0.47	0.4
nn-Unet	**0.96 (0.10)**	**0.95 (0.08)**	**0.94 (0.07)**	**0.93 (0.09)**	0.58	0.53	**9.11 (11.39)**	**7.81 (7.61)**	**9.16 (9.21)**	**10.15 (9.05)**	0.68	0.58
nvNet	0.92 (0.10)	0.93 (0.07)	0.92 (0.08)	0.89 (0.09)	0.52	0.49	12.77 (11.89)	9.27 (22.87)	13.13 (14.32)	10.44 (8.25)	0.16	0.16
**Tumor core**												
DeepMedic	0.90 (0.15)	0.82 (0.31)	0.92 (0.10)	0.71 (0.39)	0.27	0.21	30.80 (50.54)	48.22 (67.97)	9.59 (37.01)	30.53 (44.67)	0.33	0.23
nn-Unet	**0.96 (0.06)**	**0.95 (0.09)**	**0.96 (0.05)**	**0.93 (0.05)**	0.94	0.86	4.06 (8.73)	**6.00 (8.56)**	**4.58 (5.44)**	**7.63 (38.76)**	0.77	0.65
nvNet	0.94 (0.08)	0.93 (0.11)	0.94 (0.07)	0.90 (0.07)	0.86	0.78	**5.29 (8.71)**	8.54 (11.97)	5.39 (6.69)	7.74 (31.82)	0.75	0.61
**Enhancing tumor**												
DeepMedic	0.81 (0.14)	N/A	0.85 (0.10)	N/A	0.92	N/A	10.55 (23.10)	N/A	7.51 (8.01)	N/A	0.72	N/A
nn-Unet	**0.84 (0.10)**	N/A	**0.87 (0.08)**	N/A	0.77	N/A	**6.48 (8.21)**	N/A	**6.40 (6.85)**	N/A	0.8	N/A
nvNet	0.79 (0.15)	N/A	0.85 (0.10)	N/A	0.83	N/A	8.12 (6.22)	N/A	6.56 (5.70)	N/A	0.39	N/A

Bold font indicates best score in each scenario.

#### Outlier rates

For outliers according to DSC, based the IQR × 1.5 rule^42,43^, nn-Unet had the lowest outlier rate of all the models on the external test set (158 GBMs and 69 LGGs) at 3.65% of segmentations, for DeepMedic it was 8.75% and nvNet 10.28%. Outlier rates across the tumor classes were equally low for nn-Unet at 4.78% WT, 2.53% TC and 3.48% ET; DeepMedic recorded outliers at 7.88% WT, 7.24% TC, and 8.45% ET; and for nvNet 11.23% WT, 9.13% TC and 10.21% ET. A similar pattern was observed for outliers according to HD: 2.65% for nn-Unet, 36.21% for DeepMedic, and 6.78% for nvNet. Outlier rates across the tumor classes for nn-Unet at 3.58% WT, 2.56% TC and 1.37% ET. Rates for DeepMedic were 36.22% WT, 39.98% TC and 21.25% ET; and for nvNet were 8.98% WT, 8.02% TC and 5.58% ET.

## Discussion

In this study, we compared the performance of three of the top performing BraTS challenge deep learning models for automated brain tumor segmentation in an external multi-centre hospital dataset (https://www.pictureproject.nl). We extended the valuable work of the BraTS challenge by increasing the number of training cases and using a less strictly curated, and therefore more clinically-relevant, dataset^27–31^. Subsequently, we tested the generalisability of the three models on an external test set comprised of data from hospitals not used in model training. Akin to the realities of clinical assessment, we further show the utility of these models when segmenting incomplete MRI datasets, due to acquisition protocols or patient-specific circumstances, sparsified training was applied to account for missing pulse-sequences^23^. Our results demonstrate that nn-Unet, when supplemented sparsified training, produces high DSC and low HD for glioma segmentations in real-world hospital data.

### Clinical implications

Manual segmentations are the current gold standard in clinical practice, where an inter-rater variability of 0.74–0.85 DSC has been previously reported in the BraTS challenge^18,22,44^. All models’ median DSCs for both test sets were within this “clinically acceptable” inter-rater agreement range. However, manual segmentations are not a time-efficient process. Semi-automatic multi-class glioma segmentation using BrainVoyagerTM QX, ITK-Snap and 3D Slicer is reported to take an average of 18–41 min per patient^45^. On the whole, automated inference times in the current study were considerably lower than these reported semi-automated segmentation times, see Appendix 5 for all results. nn-Unet takes approximately 37 min of computer time to produce a segmentation using a CPU or only 4.5 min when a GPU is available, versus 18–41 min of human rater time.

The majority of median DSCs were within this clinically-acceptable range of 0.74–0.85^18,22,44^ when testing on an external test set with missing pulse-sequences, but there was a decrease in DSC for all models on the WT and TC, but not for the ET. The TC yielded the most accurate segmentations for both DSC and HD across models. Since the TC is the main body of the tumor and the most likely area of resection, our findings suggest that using nn-Unet with sparsified training may be an optimal combination for pre-surgical applications, with acceptable results in 97.47% of patients, based on the outlier rate of 2.53% for nn-Unet.

nn-Unet yielded the fewest outliers in all categories across all models. Furthermore, it showed the smallest reductions in segmentation performance on the external test set. There were also no statistically significant changes in segmentation quality when comparing complete versus incomplete imaging datasets. In line with other recent work, this suggests not all of the MRI sequences are necessary when models are augmented using sparsified training, or similar methods^23,46,47^. However, the lower WT DSCs indicate a heavier reliance on a full set of MRI sequences for WT segmentation, which is plausible given the hyperintensity of oedema on FLAIR and T2w. Previous studies have also used generative adversarial networks (GAN) to synthesise missing sequences with very promising results^48^, therefore direct comparison of this approach and the sparsified trained used in the current study is encouraged.

Interestingly, the nn-Unet original model used in this study came third and second in the 2017 and 2018 BraTS challenges, respectively. NvNet won the 2018 BraTS challenge but generated the lower DSCs in the current study. NvNet’s underwhelming results on incomplete datasets (Table 5) could be due to reduced effectiveness of the auto-encoder regularization in combination with sparsified training. DeepMedic won the 2017 BraTS challenge but generated the weakest HD in the current study, especially when predicting the LGG scans. The discrepancy in these findings demonstrates the value and relevance of testing models on unseen hospital-quality data with missing sequences, as we have in the current study.

### Limitations

We performed segmentations in line with the same definitions of the BraTS challenge in order to facilitate comparison, see Fig. 1. However, these definitions may not be those used in the clinical setting. In the BraTS challenge, the WT includes oedema and associated infiltrations but in reality neurosurgeons and neuroradiologists would more often classify the edge of the “tumor core” as the clinical definition of the “whole tumor”, i.e. the enhancing and non-enhancing part of the core and its associated necrosis, not including oedema. While this definition might be a better representation of the truth, current MRI techniques make it very difficult to distinguish between oedema and non-enhancing infiltrative tumor. Further research is needed to accurately distinguish between non-enhancing tumor and oedema. Depending on the intended use case for automated glioma segmentations, having a less subjective, more consistent measurement may generate a more accurate representation of true tumor infiltration, and the associated increased (inter-rater) variability. The WHO glioma classification have been updated in 2021: WHO CNS5 has some variations by further advancing the role of molecular diagnostics in the classification of CNS tumors, but remains rooted in its established methods of histology and immunohistochemistry in tumor characterisation^49^. The classification of GBM and LGG is very relevant to a model trained on combined LGG and GBM data, especially when it works on all gliomas.

Furthermore, we did not target hyperparameter optimisation for the sparsified training, nor did we make specific architecture optimisations for training and testing these models on a much larger dataset. Peak performance may be improved by doing so, but we chose not to tweak hyperparameters in order to promote generalisability.

### Future work

In our study, we have only used pre-operative scans, while post-operative and longitudinal scans are also clinically relevant for radiotherapy planning, quantitative follow-up, and automatic growth detection; however, pre-operative baseline measurements are required for these assessments. Future work should follow the BraTS challenge latest aims and include disease progression monitoring and overall survival prediction. Furthermore, the current approach relied on having at least the T1c scan available. While this is a safe assumption for most retrospective cohorts, this may be different for future cohorts due to ongoing efforts to reduce gadolinium use^50^. To support these sequences new models would have to be trained, however we have shown that sparsified training provides a simple solution to train models that are flexible to the available sequences.

The tested networks all use very different implementations, making it difficult to pinpoint which differences between the models best explain the observed performance differences. To gain a better understanding of which properties most affect performance, future development should focus on consolidating different models within a single framework and applying and testing changes gradually.

We have shown that sparsified training offers a simple solution to missing sequences that is easy to implement for different network architectures and frameworks. While dealing with missing sequences is important, and allows for the inclusion of larger (retrospective) cohorts, improving the availability of all sequences for future patients would tackle the problem at the root.

## Conclusions

In this study, we have shown the feasibility of using sparsified training alongside three top-performing BraTS challenge models to produce high-quality glioma segmentations of real-world hospital data with missing sequences. When segmenting scans with incomplete MRI sequences there was no statistically significant decrease in performance. While performance was slightly reduced in an external test set, the segmentations remained within clinically acceptable ranges. nn-Unet was the most consistent performer with highest DSCs, lowest HDs, tightest IQRs, and smallest outlier rates across the vast majority of experiments.

### Supplementary Information

Supplementary Information.

## Data Availability

The BraTS data used in this study is available through http://braintumorsegmentation.org. The PICTURE data is available from the corresponding author, upon reasonable request.

## Abbreviations

BraTS: Multimodal brain tumor segmentation
DM: DeepMedic
DSC: Dice similarity coefficient
ET: Enhancing tumor
FLAIR: Fluid-attenuated inversion recovery
GBM: Glioblastoma
HD: Hausdorff distance
IQR: Interquartile range
LGG: Low grade glioma
TC: Tumor core
VASARI: Visually AccesSAble Rembrandt Images criteria VASARI Research Project (https://wiki.cancerimagingarchive.net/display/Public/VASARI+Research+Project)
WT: Whole tumor

## Code availability

See Table 2 for links to all software availability and https://gitlab.com/picture-production/picture-qni-robust-glioma-segmentation/ for code used in this study. The nn-Unet model has also been integrated in the picture-nnunet python package https://gitlab.com/picture-production/picture-nnunet-package .
