# Supplementary material for: Multi-class glioma segmentation on real-world data with missing MRI sequences: comparison of three deep learning algorithms
Source: Sci Rep. 2023 Nov 2;13:18911. doi: 10.1038/s41598-023-44794-0 (PMC10622563; doi:10.1038/s41598-023-44794-0)
Supplement: Supplementary file 1 — Supplementary Information. [file 41598_2023_44794_MOESM1_ESM.docx]

**Supplementary Material**

**Appendix 1**

*Table A1 shows the demographic information of the patients included from the PICTURE dataset. The number of female and male patients, age range (Median with IQR), and also enhancing volume are reported.*

| Dataset | Subjects | Age  mean±IQR (range) | Sex | Tumor Core volume for GBM mean±IQR (range) |
| --- | --- | --- | --- | --- |
| Hospital1 | 104 | 38±16  (17-62) | NA | Not applied for LGG data |
| Hospital2 | 18 | 44±19 (21-65) | 7 F  11 M | Not applied for LGG data |
| Hospital3 | 4 | 56±15 (47-71) | 1 F  3 M | 33.67±19.06 ml (1.56-71.73) |
| Hospital4 | 15 | 63±21 (40-83) | 8 F  7 M | 24.72±18.76 ml (2.24-57.8) |
| Hospital5 | 22 | 59±12 (34-79) | 6 F  16 M | 26.53±46.68 ml (3.22-87.80) |
| Hospital6 | 14 | 38±18 (23-76) | 8 F  6 M | Not applied for LGG data |
| Hospital7 | 63 | 70±20 (20-84) | 30 F  33 M | 32.17±33.53 ml (2.13-142.28) |
| Hospital8 | 54 | 60±13 (20-84) | 28 F  23 M  3 NA | 36.50±43.23 ml (1.05-99.232) |
| Hospital9 | 23 | 66±13 (37-84) | 9 F  14 M | 18.34±33.15 ml (4.25-114.46) |
| Hospital10 | 8 | 63±9 (50-69) | 4 F  4 M | 34.07±22.79 ml (1.03-78.12) |
| Hospital11 | 86 | 65±16 (20-84) | 32 F  52 M  2 NA | 30.68±31.06 ml (2.89-102.45) |
| Hospital12 | 69 | 41±19 (17-74) | 30 F  38 M  1 NA | Not applied for LGG data |
| Total | 480 | 54±16 (17-84) | 163 F  207 M  6 NA | 17.54±19.68 ml (1.03-142.28) |

**Appendix 2**


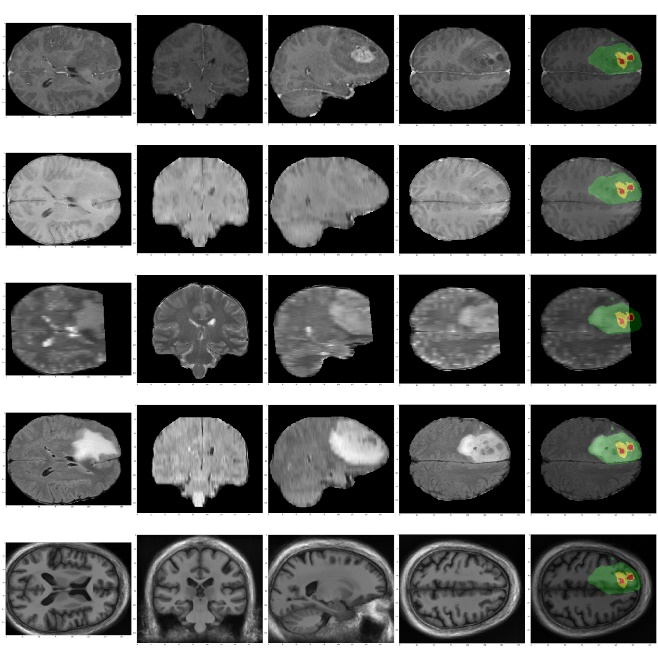

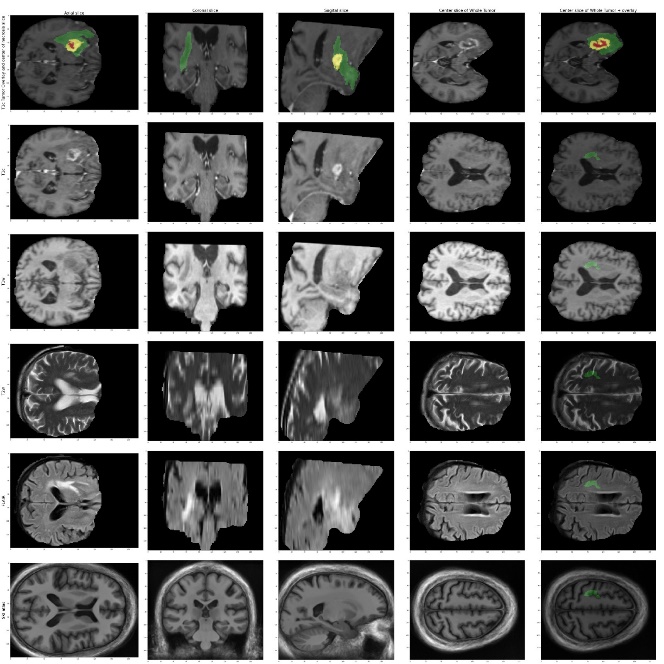


Figure A1 Left image showing an example of incomplete coverage on the T2w scan, this was not removed after quality checks. Right image showing an example of a gross registration error that was sufficiently severe to be removed from the dataset. Both images from top to bottom row: T1c, T1w, T2w, FLAIR, SRI-atlas. From left to right: 1-3: example slices in 3 directions, same for all patients to check for gross registration errors. 4-5: axial slice of the centre of the tumor, without and with the segmentation overlaid – green is WT, yellow is TC and red is necrosis.

**Appendix 3**


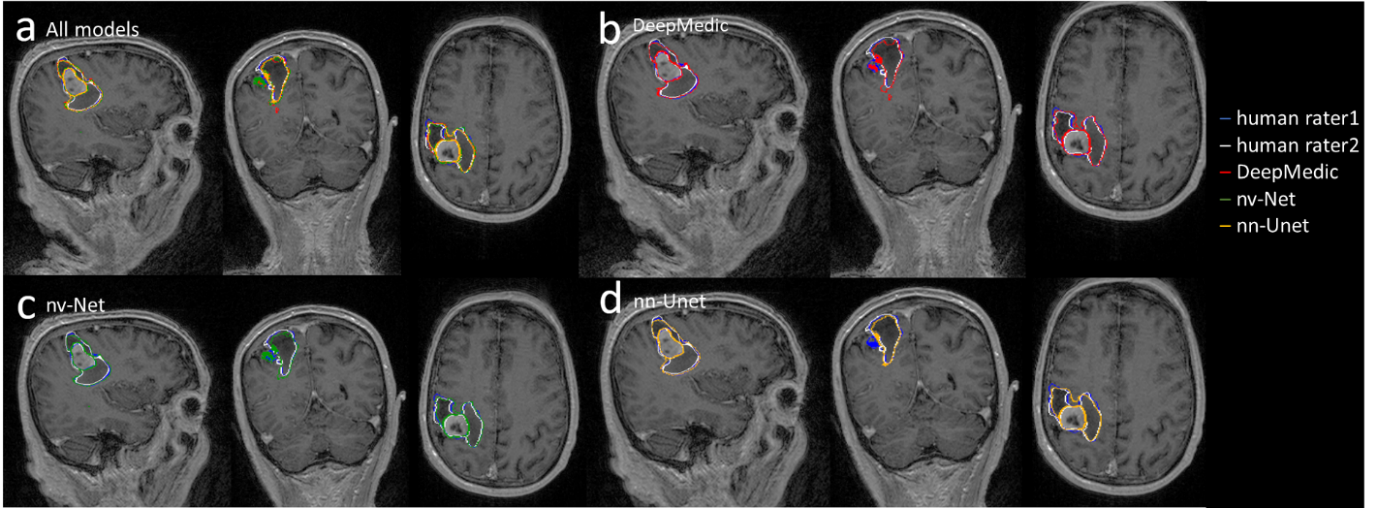


Figure A2. Shows segmentation contours on the T1c scan of a GBM patient. Image a compares two expert human raters to the automatic segmentations. The blue and white contours are two human experts’ manual segmentation of both WT and ET, the red contour is generated by DeepMedic, green contour by nv-Net, and orange by nn-Unet. The other images highlight how the different algorithms perform on this scan, compared to the inter-rater variability. b-d show each algorithm plus the segmentations by two human experts, b – DeepMedic, c – nv-Net, d – nn-Unet.

**Appendix 4**


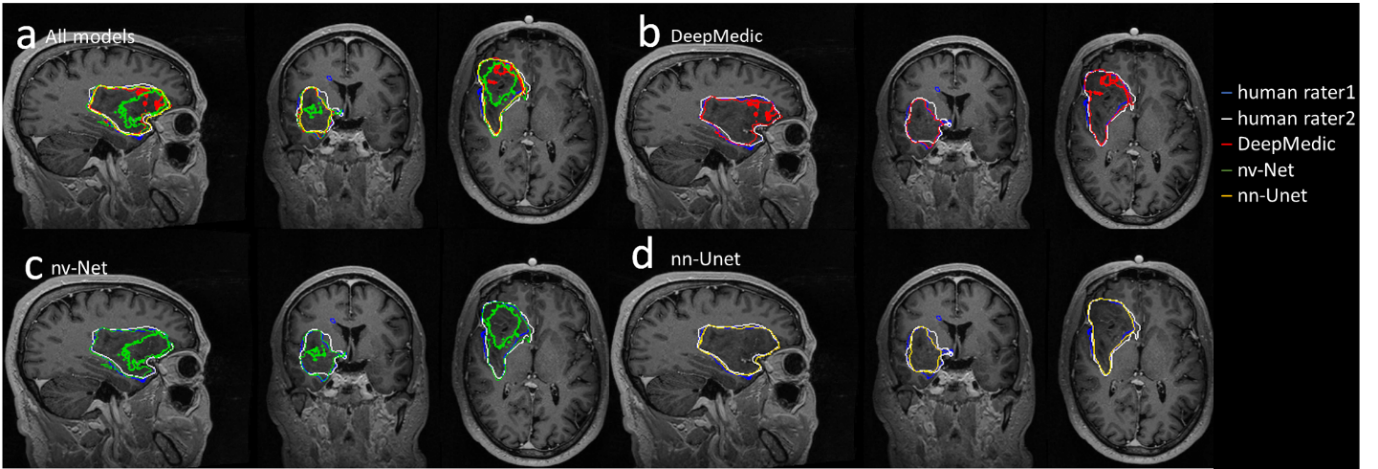


Figure A3. Shows segmentation contours on the T1c scan of an LGG patient. Image a compares two expert human raters to the automatic segmentations. The blue and white contours are two human experts’ manual segmentation of both WT and ET, the red contour is the DeepMedic, green contour is nv-Net, and orange is nn-Unet’s output. The other images highlight how the different algorithms perform on this scan, compared to the inter-rater variability. Images b-d show each algorithm plus the segmentations by two human experts, b – DeepMedic, c – nv-Net, d – nn-Unet.

**Appendix 5**

**Training and Inference Time**

Training times for the whole dataset were shortest for DeepMedic (~18 hours), followed by nvNet (~27 hours) and the longest by some distance was nn-Unet at 23.7 days, which consisted of training the 2D model for 4.6 days and the 3D part for 19 days. Pre-processing and inference times of a single patient on both central (CPU) and GPU are shown in Table A2.

Table A2 pre-processing and inference times in seconds for each model on both central (CPU) and graphics processing units (GPU).

| **Model** | **Pre-processing**  **time (s)** | | **Inference time (s)** | | **Total time (s)** | |
| --- | --- | --- | --- | --- | --- | --- |
|  | **CPU** | **GPU** | **CPU** | **GPU** | **CPU** | **GPU** |
| **DeepMedic** | 390 | 202 | 170 | 32 | 560 | 234 |
| **nv-Net** | 390 | 202 | 17 | 15 | 407 | 217 |
| **nn-Unet** | 390 | 202 | 1860 | 72 | 2250 | 274 |
